# Surgical treatment of rectal cancer with a Retzius shunt: a case report

**DOI:** 10.1186/s40792-019-0583-z

**Published:** 2019-02-18

**Authors:** Toshinori Sueda, Mitsuyoshi Tei, Haruna Furukawa, Tae Matsumura, Chikato Koga, Masaki Wakasugi, Hiromichi Miyagaki, Ryohei Kawabata, Junzo Shimizu, Atsuya Okada, Junichi Hasegawa

**Affiliations:** 1 Department of Surgery, Osaka Rosai Hospital, 1179-3 Nagasonecho, Kita-ku, Sakai, 591-8025 Japan; 2 Department of Diagnostic and Interventional Radiology, Osaka Rosai Hospital, 1179-3 Nagasonecho, Kita-ku, Sakai, 591-8025 Japan

**Keywords:** Venous malformation, Retzius shunt, Rectal cancer

## Abstract

**Background:**

A case of a short circuit (Retzius shunt) from the inferior mesenteric vein (IMV) to the inferior vena cava (IVC) without accompanying portal hypertension due to liver cirrhosis is rare.

**Case presentation:**

An 83-year-old woman who was followed after surgery for thyroid and breast cancer was incidentally found to have rectal cancer on computed tomography (CT). Preoperative three-dimensional CT showed a venous malformation forming a short circuit (Retzius shunt) from the IMV to the IVC. Laparoscopic anterior rectal resection was performed. Operative findings included the Retzius vein crossing the abdominal aorta and the inferior mesenteric artery (IMA) to the IVC and a number of engorged vessels in the mesentery. The Retzius vein and IMA were clipped without major bleeding, and tumor-specific mesorectal excision was then performed. The patient’s postoperative clinical course was good, and she was discharged without complications.

**Conclusions:**

Preoperative imaging enabled identification of an unexpected rare disease, thus reinforcing the importance of preoperative imaging.

## Background

The veins of Retzius are rare and may be primary (congenital or idiopathic) or secondary due to portal hypertension or trauma, or iatrogenic. A case of a short circuit (Retzius venous short circuit) from the inferior mesenteric vein (IMV) to the inferior vena cava (IVC) without accompanying portal hypertension due to liver cirrhosis is extremely rare [1–3]. The veins of Retzius are important, because they can be injured during surgery, and they may provide a pathway for the hematogenous spread of colorectal cancer.

A case in which an unexpected Retzius venous short circuit between the IMV and IVC was discovered during three-dimensional computed tomography (3D-CT) prior to surgery is presented. Using this information, laparoscopic anterior resection was safely performed. Preoperative 3D-CT is useful for understanding the anatomy to ensure a safe, precise operation. The present case study highlights the importance of preoperative imaging.

## Case presentation

**Fig. 1:**
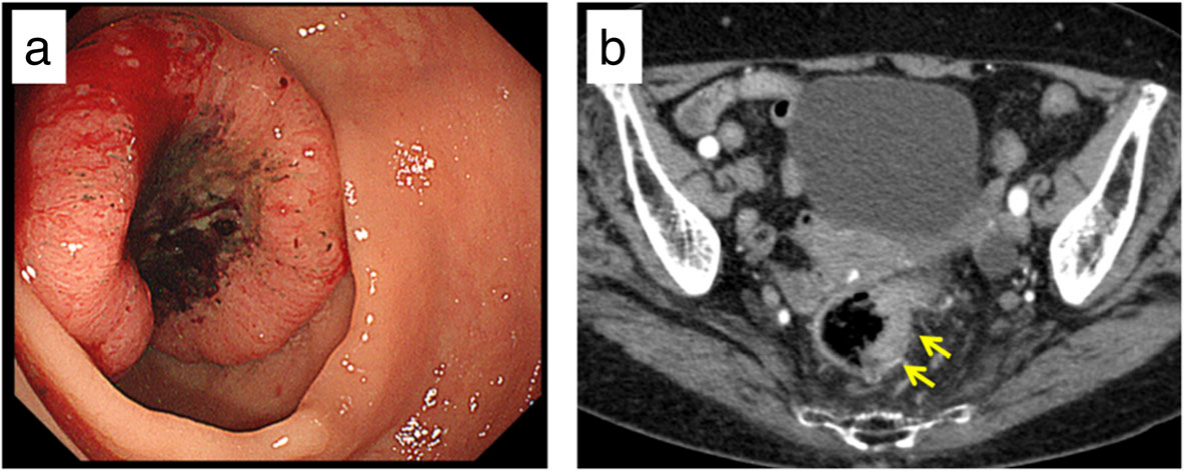
Preoperative findings. **a** Preoperative colonoscopy shows a type 2 tumor localized in the upper rectum. **b** An abdominal contrast CT scan shows wall thickening of the rectum (yellow arrows)

**Fig. 2:**
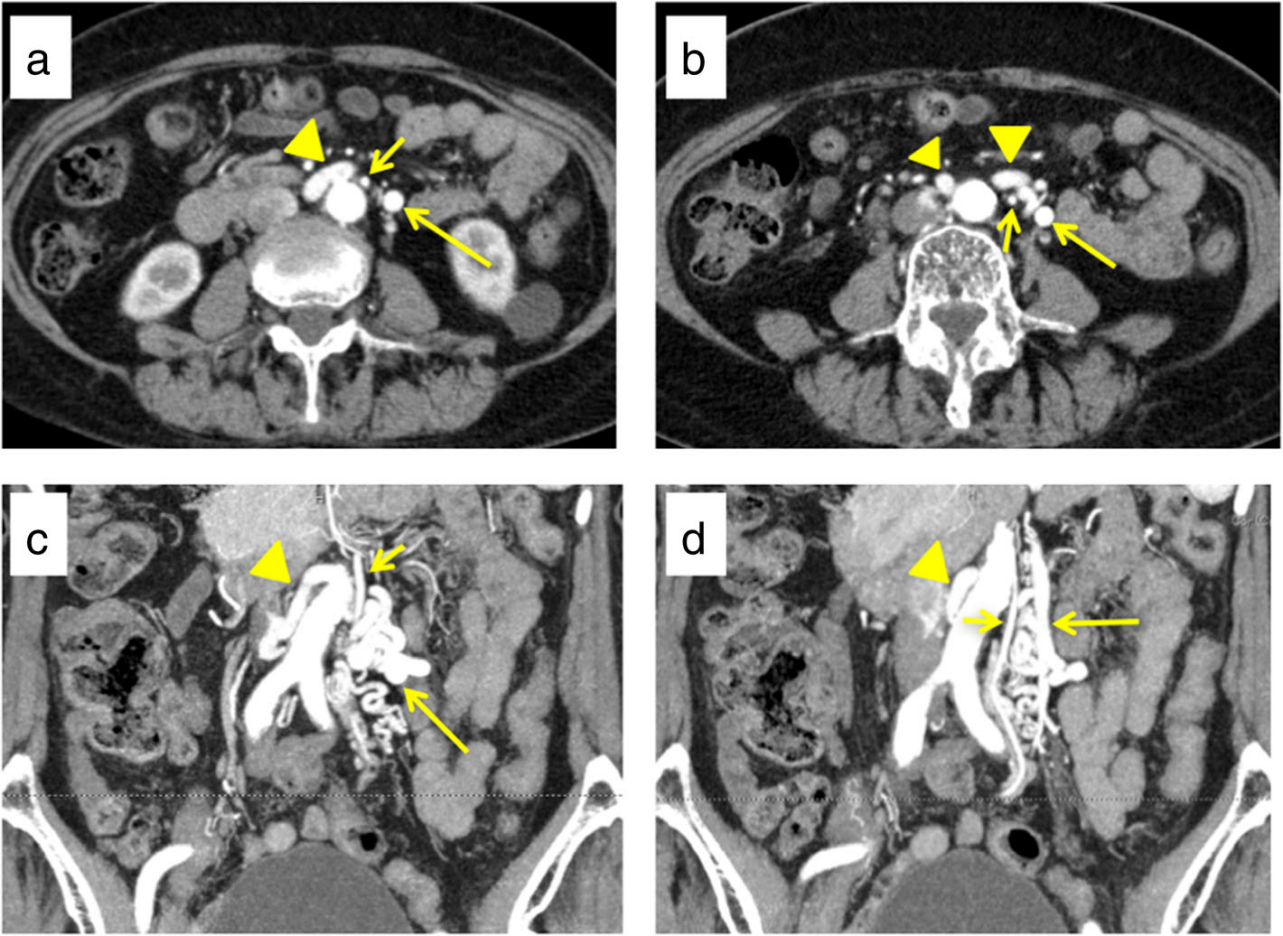
An abdominal contrast CT scan shows a venous malformation forming a short circuit (Retzius shunt) from the IMV to the IVC. **a**, **b** Axial views. **c**, **d** Coronal views. Retzius shunt (arrow head). IMV (long arrow). IMA (short arrow)

**Fig. 3:**
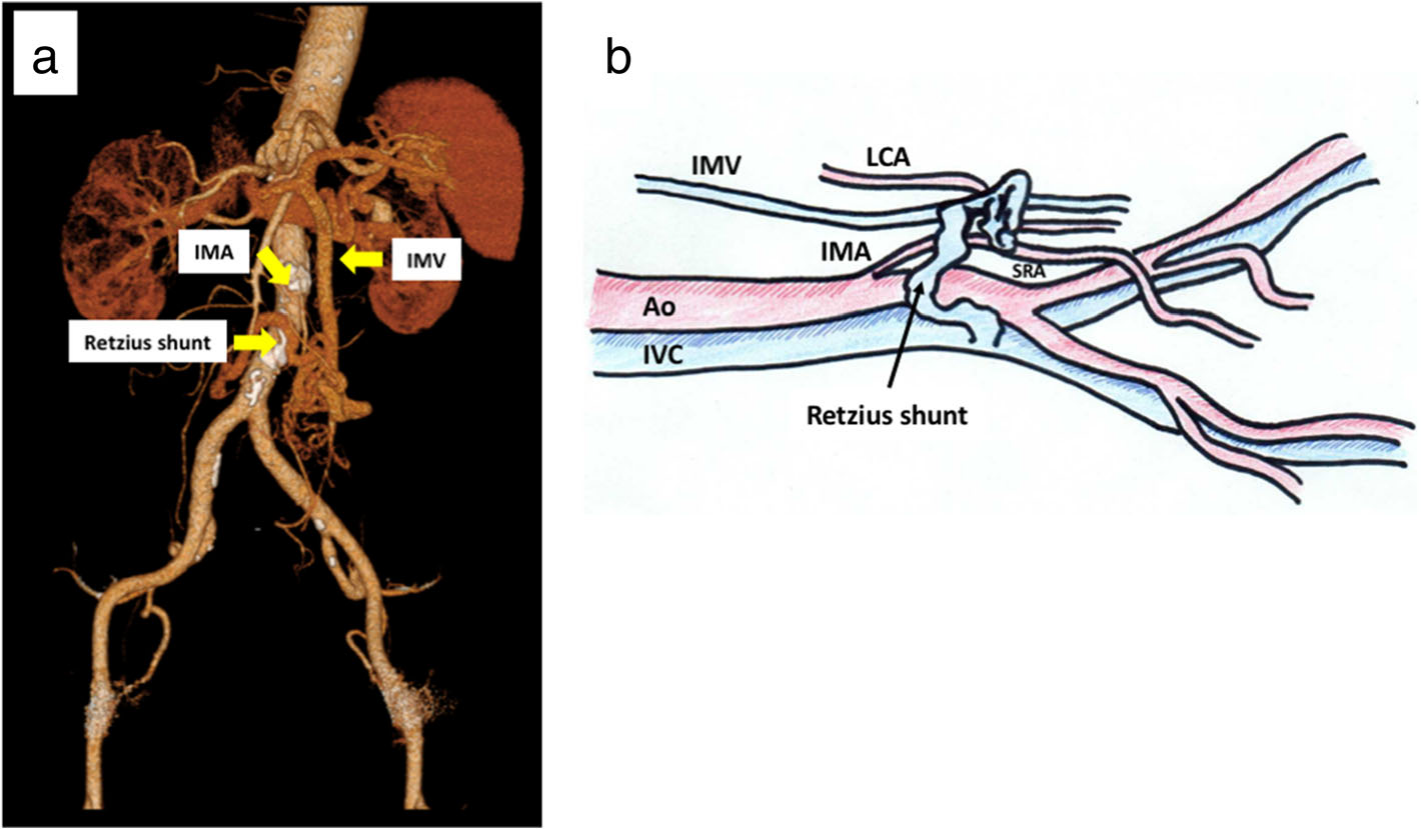
Preoperative 3D-CT scan of vessels shows dilated veins in the inferior mesenteric area and the Retzius vein crossing the abdominal aorta and IMA to the IVC. **a** Frontal view. **b** Lateral view (hand-drawn picture)*.* IMA inferior mesenteric artery, IMV inferior mesenteric vein, IVC inferior vena cava, LCA left colic artery, SRA superior rectal artery, Ao abdominal aorta

The case of an 83-year old woman with a history of thyroid cancer, breast cancer, and rheumatoid arthritis is presented. She had no relevant family history. After surgery for thyroid and breast cancers, elevations of carcinoembryonic antigen and carbohydrate antigen 19-9 were observed. She had no abdominal tenderness, and no mass was palpable. Laboratory results were unremarkable. Colonoscopy showed a type 2 tumor localized in the upper rectum (Fig. 1a). Following biopsy, the lesion was confirmed to be moderately differentiated adenocarcinoma. Contrast CT examination showed wall thickening of rectal cancer and swollen lymph nodes, but there were no distant metastases (Fig. 1b). In addition, abdominal contrast CT examination also revealed vascular anomaly (Fig. 2a–d). Laparoscopic surgery was planned, and a 3D-CT was constructed from contrast CT images to investigate local vascularity. The 3D-CT scan showed a venous malformation forming a short circuit (Retzius shunt) from the IMV to the IVC (Fig. 3a, b).

**Fig. 4:**
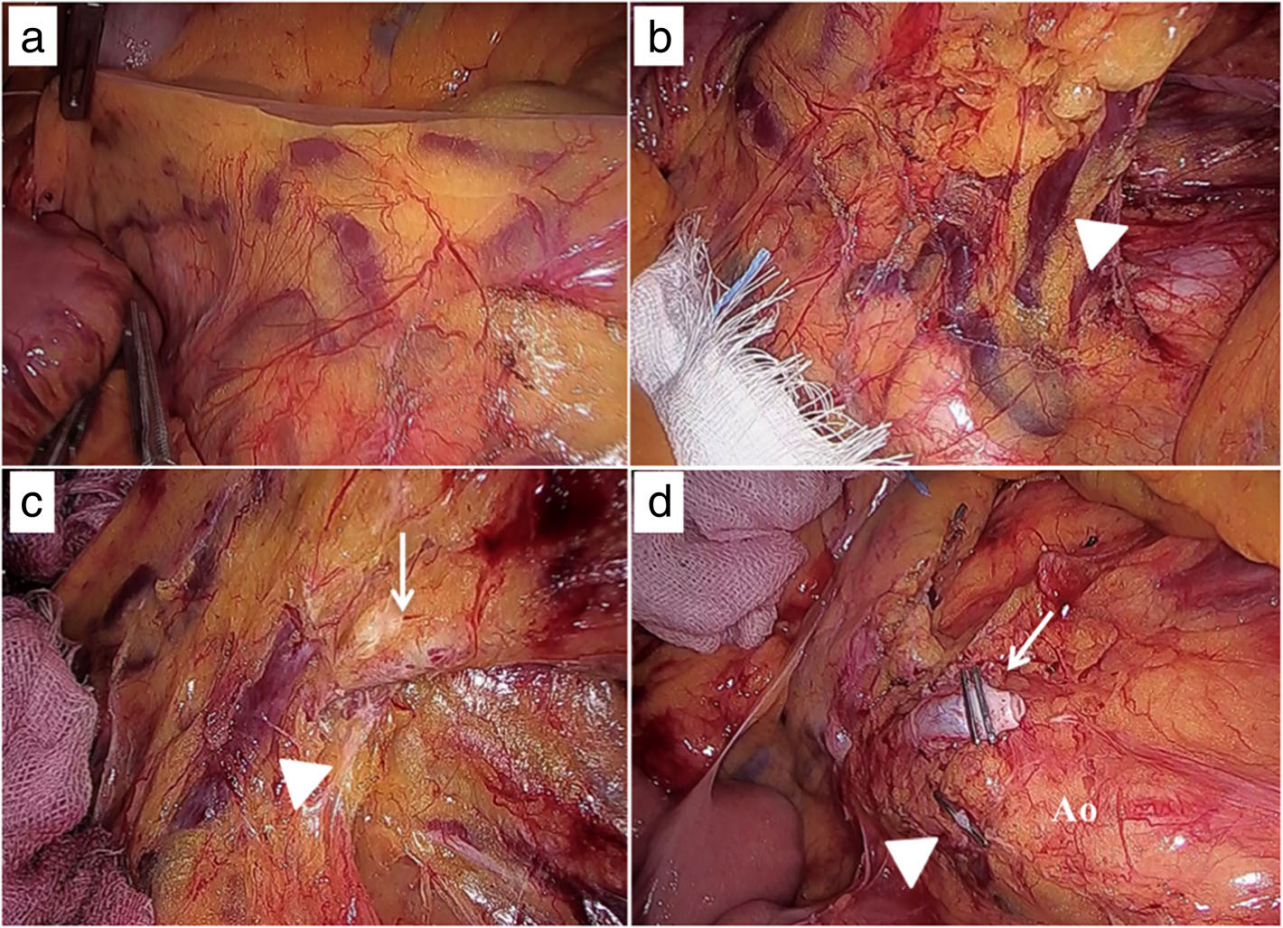
Surgical view. **a** Engorged vessels in the mesentery. **b**, **c** Retzius vein (arrowhead) crossing the abdominal aorta (Ao) and IMA (arrow) to the IVC. **d** Retzius veins (arrowhead) and the IMA (arrow) are ligated with vascular clips

Based on these findings, upper rectal cancer with a Retzius shunt from the IMV to the IVC was diagnosed. Laparoscopic anterior resection was performed. Laparoscopic observation showed a number of engorged vessels in the mesentery (Fig. 4a) and the Retzius vein crossing the abdominal aorta and inferior mesenteric artery (IMA) to the IVC (Fig. 4b, c). The Retzius vein and IMA were clipped without major bleeding (Fig. 4d), and then tumor-specific mesorectal excision was completed. The patient was discharged on the 14th day after surgery with no complications. Histological examination showed the tumor to be moderately differentiated adenocarcinoma with invasion of the subserosa (T3) and lymph node metastasis (N2). No distant metastases were found (M0) at the time of surgery. The histological TNM staging of the tumor was stage IIIB, with no other remarkable findings.

## Discussion

The present case highlights the usefulness of preoperative examination. Careful preoperative examination showed a rare venous malformation, which reduced the stress of the surgeons and improved operative accuracy.

Retzius reported cases of a short circuit from the duodenum to the IVC and from the left colon to the left renal vein in 1835 [1]. The retroperitoneal intestinal vein-general circulation anastomotic pathway is now called the Retzius vein [2, 3]. The cause of a Retzius venous shunt is not completely understood; however, two major theories have been proposed [3–7]. The first is the congenital origin theory, which suggests persistence of the communication between the portal and caval systems that occurs during embryonal development. The second is the acquired theory, which suggests that the shunt results from trauma, portal hypertension by liver cirrhosis, etc. A Retzius venous shunt usually occurs in patients with portal hypertension and is mainly induced by liver cirrhosis, but it can sometimes occur in patients who do not have liver cirrhosis. In fact, a previous imaging study showed that the veins of Retzius were demonstrated on CT arterial portography in approximately 50% of patients with and 50% of patients without liver cirrhosis [8]. The presently described patient had no liver cirrhosis or history of trauma. Thus, the Retzius venous shunt in the present case was thought to be congenital in origin.

Preoperative risk assessment is an important aspect of surgical planning because it helps surgeons identify patients with an increased risk of a poor postoperative outcome [9]. CT imaging is widely available, and all patients with cancer routinely undergo preoperative CT for staging. Recently, 3D-CT was shown to help in the preoperative assessment of vascular anatomy for laparoscopic lymph node dissection. The 3D-CT is a combined technology of a conventional CT scan with that of traditional angiography to create detailed images of vessels in the body, and noninvasive and provides highly reliable and reproducible vascular anatomy. Furthermore, 3D-CT also allows noninvasive vascular assessment and is used widely in preoperative planning workups for patients requiring laparoscopic surgery and in the evaluation of vascular anomaly and other vascular conditions [10]. When the preoperative 3D-CT is obtained for these purposes, it can simultaneously serve as a tool for preventing a serious situation, such as an unexpected bleeding or conversion to open surgery, during laparoscopic surgery. In fact, several authors reported that imaging of vascular anatomy with 3D-CT facilitates surgery [11–19]. We also use 3D-CT constructed from routine contrast CT images to preoperatively evaluate the tumor-feeding artery, drainage vein, and vascular anatomy. In the present case, 3D-CT unexpectedly showed a Retzius venous short circuit between the IMV and IVC. Laparoscopic surgery with a Retzius venous short circuit would have been dangerous without the detailed preoperative examination, but the preoperative information acquired by 3D-CT made the surgery the safest operation possible. Based on this case report, we would like to stress the importance of recognizing the veins of Retzius which, if unrecognized, could lead to significant vascular complications, including hemorrhage. As the resolution of CT increases, the importance of preoperative imaging for patients with vascular malformations will be even greater in the future.

## Conclusions

In the present case, preoperative imaging enabled the discovery of an unexpected rare venous malformation. The information obtained from this preoperative examination was sufficient to enable adequate safety precautions and to perform curative resection with laparoscopic surgery, without any complications. The present case re-affirms the importance of preoperative diagnosis for patients with vascular abnormalities.

## Abbreviations

3D-CT: Three-dimensional computed tomography
Ao: Abdominal aorta
CT: Computed tomography
IMA: Inferior mesenteric artery
IMV: Inferior mesenteric vein
IVC: Inferior vena cava
LCA: Left colic artery
SRA: Superior rectal artery
